# Carvacrol and thymol effect in vapor phase on *Escherichia coli* and *Salmonella* serovar Typhimurium growth inoculated in a fresh salad

**DOI:** 10.1016/j.heliyon.2024.e29638

**Published:** 2024-04-16

**Authors:** Teresa Soledad Cid-Pérez, Ricardo Munguía-Pérez, Guadalupe Virginia Nevárez-Moorillón, Carlos Enrique Ochoa-Velasco, Addí Rhode Navarro-Cruz, Raúl Avila-Sosa

**Affiliations:** a Departamento de Bioquímica-Alimentos, Facultad de Ciencias Químicas, Benemérita Universidad Autónoma de Puebla, Mexico; b Laboratorio de Micología, Centro de Investigaciones en Ciencias Microbiológicas, Instituto de Ciencias, Benemérita Universidad Autónoma de Puebla, Mexico; c Facultad de Ciencias Químicas, Universidad Autónoma de Chihuahua, Circuito Universitario S/n Campus, Universitario II, Chihuahua, 31125, Mexico

**Keywords:** Vapor phase, Natural antimicrobials, Active packaging

## Abstract

This study aimed to evaluate the antimicrobial effect of thymol and carvacrol in inhibiting *Escherichia coli* and *Salmonella* serovar Typhimurium inoculated on a fresh green salad through the vapor phase. A film-forming solution was prepared by dissolving starch, sorbitol, and variying concentrations of carvacrol, thymol, and a mixture of both. The film-forming solution containing the respective antimicrobial agent was then added lid, which was sealed rigidly and hermetically to achieve different concentrations (105 mg/L of air of carvacrol, 105 mg/L of air of thymol, and a mixture of 52 mg/L of air of carvacrol and 52 mg/L of air of thymol). Each active package contained fresh green salad inoculated with *E. coli* or *Salmonella* serovar Typhimurium. The active packages were then sealed and refrigerated at a temperature of 6 °C for 48 h. Growth/inhibition curves were modelled using the Weibull equation, and consumer acceptance was evaluated. Carvacrol can reduce up to 0.5 log-cycles, while thymol can reach almost 1 log cycle. Blending the components with half the concentration has a synergistic effect, inhibiting up to 2.5 log cycles. Consumer ratings revealed no significant differences between the packages. However, the average score was 5.4 on a 9-point hedonic scale, evaluators' comments did not indicate dislike or a strong taste characteristic of thymol and carvacrol.

## 1 Introduction

Leafy vegetables, which are often consumed raw, can be associated with outbreaks of foodborne pathogens such as *Escherichia coli*, *Listeria,* and *Salmonella* [1,2]. During postharvest processing, processors rely on cleaning and sanitizing methods to reduce the microbial load of the product, maintain its quality, extend its shelf life, and ensure its microbial safety [1,[3], [4], [5]]. Fresh-cut or ready-to-eat (RTE) vegetables are the fastest-growing segment in the vegetable industry today. Additionally, RTE vegetable salads are highly popular in developing countries [6,7]. This worldwide success can be attributed not only to the freshness and nutritional value of the product but also to its convenience [3,8,9]. Significant efforts have been made to enhance the microbiological safety of RTE salads. Various techniques have been employed to control microbial pathogens, including the use of disinfectants, optimization of atmospheric and storage conditions, and the implementation of innovative processing techniques to meet market demand [1,6,10,11].

One particularly promising technique is active food packaging, which involves incorporating different types of agents into the packaging materials. This active food packaging interacts chemically or biologically with various components of the packaging to preserve freshness and ensure the safety of the food product [12]. These systems contain components that can be released into or absorbed from the packaged food or its environment. The aim is to increase the required amount of active substances with antimicrobial and/or antioxidant properties by adding them to the packaging material instead of directly adding them to the food. These active agents are usually obtained from natural compounds [13]. Usually, they reduce or delay the growth of microorganisms [[14], [15], [16]] and can obstruct the passage of water vapor, oxygen, and carbon dioxide from the environment [17]. Furhtermore, consumers demand healthier, more natural products, as well as practical features such as RTE and longer shelf life, even under adverse conditions. Antimicrobial packaging fulfills these requirements by delivering active ingredients onto the food surface in a controlled manner [[18], [19], [20]].

Essential oils (EOs) have been proven to be highly effective against various food microorganisms [21,22]. They are particularly effective in the vapor phase, as they can produce low-molecular-weight or volatile molecules [5,[23], [24], [25]]. In situations where direct contact is not possible, such as with large areas or products, Eos can still be effective. One advantage of using antimicrobial molecules in the vapor phase is that they can reach the entire food [26]. These molecules are gradually released into the packaging, providing a constant antimicrobial effect. Using the antimicrobial agent as a vapor inside the packaging is often preferred because it minimizes direct interaction between the packaging material and the food product, resulting in fewer changes to sensory and physicochemical properties [27]. In addition, the trends in food preservation indicate the use of natural antimicrobial mixtures to ensure food safety and quality at lower concentrations of antimicrobials [14]. These mixtures offer a wider range of enhanced activity against various pathogenic microbes or target multiple sites within the cell, making them easier to control compared to single agents [28].

One of the most effective natural antimicrobials is thymol and carvacrol, which are phenolic compounds present in oregano and thyme EOs. These compounds have been shown to significantly inhibit the growth of both gram-positive and gram-negative bacteria, as well as viruses and fungi. Thymol and carvacrol are generally considered safe and the have been utilized in oral health, as confirmed by their use as food flavorings, and are also considered antimicrobial and antioxidant food additives [29,30]. Several studies have reported the incorporation of carvacrol and thymol into various materials such as polylactic acid, polycaprolactone, polypropylene, or polyethylene [[31], [32], [33], [34]]. In most of those studies, traditional methods, solvent casting, melt blending, or compression molding were used to load thymol and carvacrol into different polymer films. However, all of these techniques suffer from certain drawbacks, such as evaporation, thermal degradation, or uneven distribution of the active substance, as well as poor mechanical properties of films [13,35]. The mixture of thymol and carvacrol was reported to have positive synergetic antimicrobial effects. The use of synergistic combinations allows for achieving the desired antimicrobial and antioxidant performance at lower concentrations of the active components, which is very important for the safety of thymol and carvacrol when used in active food packaging [13,30].

Finally, despite the potential antimicrobial activity of thymol and carvacrol, there are some limitations to their use as free agents, such as their high volatility, hydrophobicity, and degradation in water [30]. Moreover, Rojas et al. [35] highlighted important challenges such as determining the optimal processing technique and developing strategies for incorporating active compounds while preventing their loss and degradation. Their incorporation into foods by vapor phase can affect some sensory perception properties and stability during storage [36]. Several studies have demonstrated the antimicrobial activity of EOs in the vapor phase [18,[37], [38], [39], [40], [41], [42], [43]]. However, there are few studies on the antimicrobial activity of active packaging films containing EOs derivative molecules in the vapor phase, confirming such activities should be performed on actual food products owing to the complexity of microbial flora and food matrix [15,23,26,30].

The aim of this work was to evaluate the antimicrobial activity of thymol and carvacrol in inhibiting *E. coli* and *Salmonella* serovar Typhimurium inoculated into a fresh green salad via the vapor phase by i) incorporating thymol and carvacrol into an active package via edible starch films; ii) modeling the inhibitory activity of thymol, carvacrol, and their combination; and iii) evaluating whether the vapor phase of thymol, carvacrol, and their combination affects the sensory profile of RTE salad.

## 2 Materials and methods

### 2.1 Biological material, chemical reagents, and culture media

Roman lettuce (*Lacttuca scariola* L. var. longifolia) and spinach (*Spinacia oleracea*) were obtained in March 2018 from a local market in Puebla, Mexico. The leaves were washed with tap water to remove foreign matter. Then, they were sliced and disinfected with chlorinated water (100 mg/L). After disinfection, they were rinsed and drained to remove as much water as possible and eliminate the residual effect of NaOCl from the surface of the salad.

*Escherichia coli* (ATCC 25922) and *Salmonella* serovar Typhimurium (ATCC 14028) were obtained from the collection of the Facultad de Ciencias Químicas (Benemérita Universidad Autónoma de Puebla). Strains were maintained in a brain-heart infusion medium. For the preparation of fresh inoculum, microorganisms were grown to the exponential phase for 18 h at 37 °C in nutrient broth.

Reagents (Thymol and carvacrol: product numbers W306606 and W224502, respectively) were obtained from Sigma-Aldrich, Inc. (Toluca, Mexico), and the culture media used in this study were from Merck (Mexico City, Mexico).

### 2.2 Preparation of active packaging

#### 2.2.1 Incorporation of antimicrobial substances

**Fig. 1:**
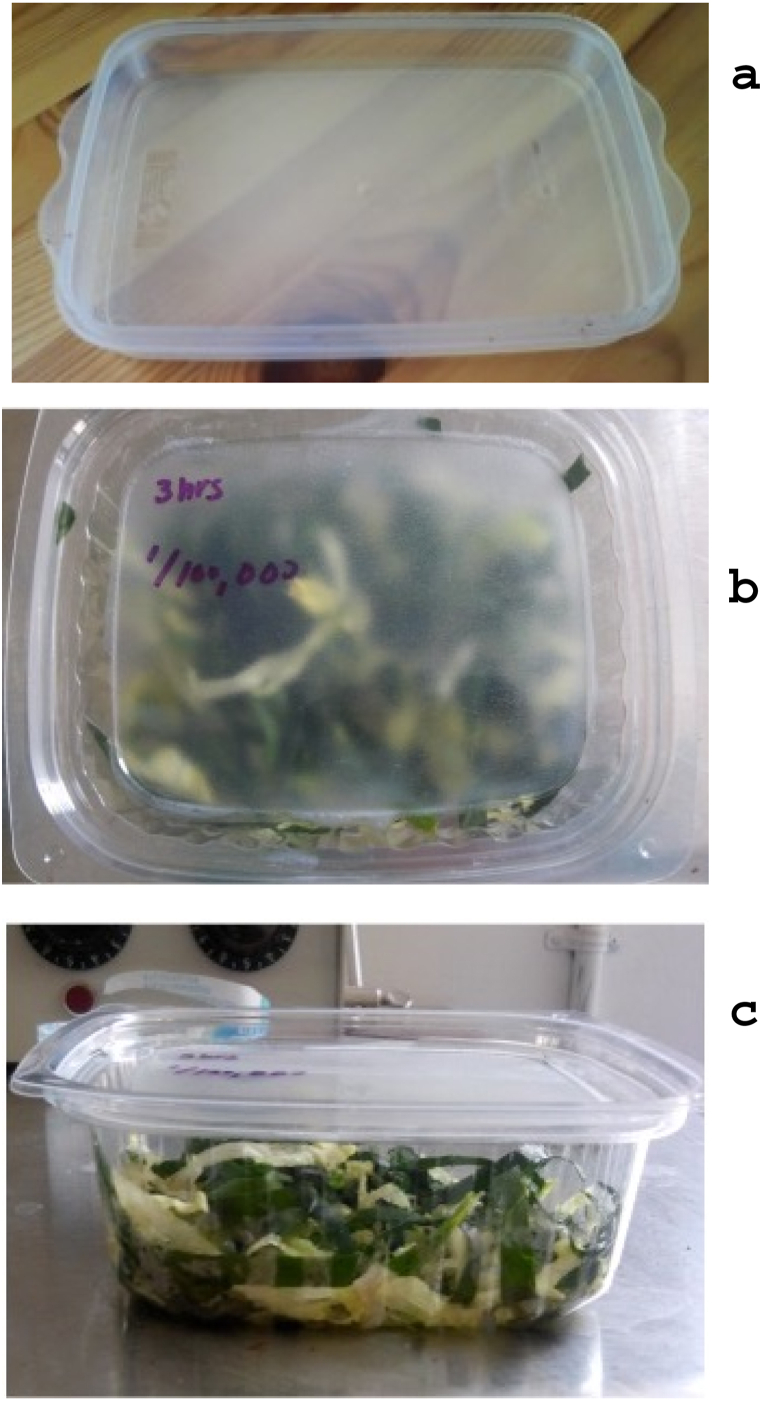
Active packaging system design a) lid with the film-forming solution; b) lid with the edible film added to rigid hermetically sealed packages; c) active package sealed and stored.

To incorporate natural antimicrobial substances, a film-forming solution was prepared by dissolving starch (1 % w/w) and sorbitol powder (1 % w/w) in 0.125 N sodium hydroxide solution. Four film-forming solutions were prepared, to which the antimicrobial substances were added [44]. Carvacrol and thymol were each added at a total concentration of 0.99 % w/w, comprising 0.495 % w/w each. The fourth control was a film-forming solution alone, without any antimicrobial agent, while the second control contained neither film nor antimicrobial agent [45]. Three ml of the film-forming solution containing the respective antimicrobial agent were added to the lids of rigid, hermetically sealed packages and allowed to dry until an edible film was formed. This procedure was performed three times to achieve the formation of layers (Fig. 1A). Three layers of 1 mL of the film-forming solution were applied to each pack. The following formula was applied to calculate the final concentration of the antimicrobials: For the 0.99 % w/w concentrations, there was approximately 30 mg/L of thymol or carvacrol. Each pack had an air volume of 0.856 L. Applying the formula C_1_V_1_=C_2_V_2,_ the concentration was 0.105 mg/mL of air, or 105 mg/L of air.

#### 2.2.2 Packaging and storage

Each active package contained 32 g of fresh green salad (Fig. 1B) inoculated with *E. coli* or *Salmonella* serovar Typhimurium at a concentration of 0.5 (1.5 × 10^8^ CFU/mL) on the McFarland scale using microdispersion. The active packages were then sealed and refrigerated at a temperature of 6 °C for 48 h (Fig. 1C). To determine the microbial load of the salad, 1 g of lettuce was sampled, and the aerobic plate count was determined at different storage times (0, 3, 6, 9, 12, 24, and 48 h). Sampling was done in triplicate by taking 1 g of lettuce from each variant under study and placing it on a plate with nutrient agar that was incubated at 37 °C for 24 h.

### 2.3 Modeling microbial growth/inhibition

Growth or inhibition curves were generated from experimental data by plotting log(*N/No*), where *N* was the average number of CFU/g at time (*t*) and *No* was the average number of CFU/g at the initial time point. Microbial growth or inactivation curves were fitted using the Weibull model (Eq. (1)) through nonlinear regression (KaleidaGraph 3.51, Synergy Software, Reading, PA, USA)

(1) $$\log\frac{N}{N_{o}}=-bt^{n}$$

* where *b* and *n* are the scale and shape parameters of the model, respectively.

The Weibull model corresponds to an upward concave survival curve when *n* > 1, a downward concave curve when *n* < 1, and a linear curve when *n* = 1. This results in two parameters: *b*, which indicates the rate of bacterial inactivation, and *n*, which indicates how the microorganisms survive or die.

The parameters *n* and *b* were used to calculate the frequency distribution of resistance. The mode of distribution represents the treatment time at which most of the population dies or is inactivated. The mean value represents the average inactivation time with its variation. Once the values of *b* and *n* were determined, the resistance frequency curves were plotted using the following equation (Eq. 2):

(2) $$\frac{d\Phi }{dt_{0}}=bnt_{c}^{n-1}\;\exp\;\left(-bt_{c}^{n}\right)$$

* where *t*_*c*_ is a measure of the resistance or sensitivity of the organism, and *Φ* is the fraction of organisms that divide at a given time [46].

### 2.4 Consumer evaluation

Consumer acceptability was assessed using a 9-point hedonic scale [47]. The scale ranged from 1, indicating strong dislike, to 9, indicating strong agreement. Twenty g of uninoculated lettuce from the three combinations were packaged and stored with a control. The test was performed with one hundred untrained judges (students aged 18–25) from Benemérita Universidad Autónoma de Puebla (Puebla, Mexico) who evaluated taste, odor, color, texture, and general acceptability. Tests were conducted after 0, 6, and 12 days of storage.

### 2.5 Statistical analysis

All determinations were performed in triplicate, and each experiment was performed twice. The results were analyzed through analysis of variance using Minitab v.16 software (Lead Technologies Inc., USA). Statistical differences between results were determined using Tukey's comparison test (α = 0.05).

## 3 Results and discussion

### *3.1* *E. coli* and *Salmonella serovar* Typhimurium inhibition

**Fig. 2:**
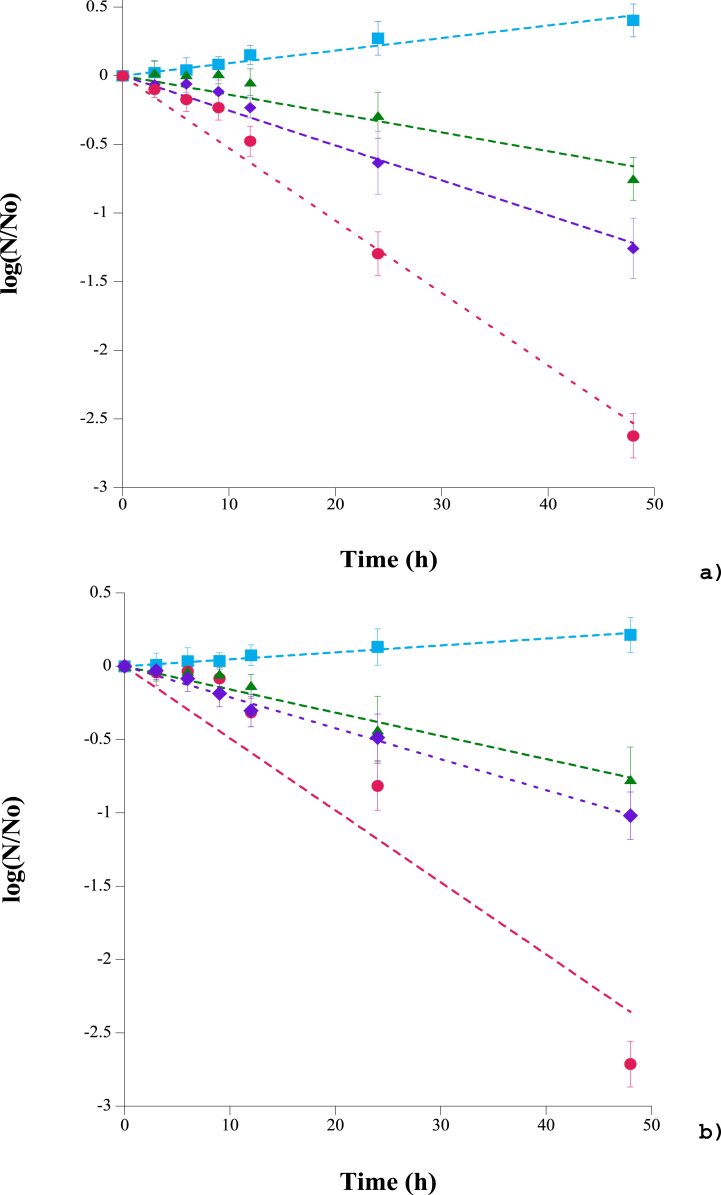
Effect of carvacrol, thymol and their combinations in vapor phase on growth/inhibition curves of **a)***Escherichia coli* and **b)***Salmonella* serovar Typhimurium inoculated in a fresh salad during 48 h at 6 °C in different active packaging systems (Control , 105 mg/L of air of carvacrol , 105 mg/L of air of thymol , and a mixture of 52 mg/L of air of carvacrol and 52 mg/L of air of thymol ).

Fig. 2 shows the behavior of *E. coli* (Fig. 2A) and *Salmonella* (Fig. 2B) inoculated in a fresh salad. It can be observed that in both control curves, the number of microorganisms remains constant and even slightly increases in the period between 24 and 48 h. The storage temperature of the salads (5-7 °C) was not sufficient to achieve the inhibition of both microorganisms. Firmanda et al. [19] pointed out that products with high water content are an excellent medium for proliferation of pathogenic microorganisms. Such contamination can accelerate changes in sensory quality and even pose the risk of disease. Liao & Wang [4] reported that the level of *E. coli* in lettuce inoculated on day 0 increased by about 0.9 log from day 5 to day 10. Furthermore, Osaili et al. [9] reported that improper distribution kinetics in addition to inadequate refrigeration during lettuce storage can compromise its microbiological quality and safety. An increase in aerobic and mesophilic microbial counts was observed in lettuce samples when the best-before date was reached [23].

On the other hand, the inhibitory effect of thymol, carvacrol, and their mixture can be observed in the vapor phase (Fig. 2). In general, carvacrol can reduce up to 0.5 log cycles, while thymol nearly achieves a full cycle reduction. In most of the in vitro experiments with these types of compounds [48], it was observed that the inhibition increases with increasing concentration of the compound. Yammine et al. [49] reported the effect of free and nanoencapsulated thymol and carvacrol on *Salmonella* Enteritidis biofilms adhered to stainless steel. Eradication of biofilms developed on stainless steel was achieved following a 15-min treatment with nanoencapsulated antimicrobials at values near 0.62 and 0.31 mg/L of thymol and carvacrol, respectively.

It is observed that a mixture with half the concentration of the components has a synergistic effect, capable of inhibiting up to 2.5 logarithmic cycles. This effect was attributed to the cumulative sub-lethal damages caused by the two antimicrobials, capable of hindering possible cell repair [50]. Moreover, Arioli et al. [51] highlighted that carvacrol damages bacterial cell membranes more efficiently, affecting membrane integrity. The presence of a free hydroxyl group, which can act as a transmembrane proton carrier, is important for carvacrol's antimicrobial activity. Thus, the increasing membrane permeability after exposure to thymol and carvacrol reﬂects the damage of cell membranes induced by terpenes, which act as substitutional impurities in the phospholipid bilayer [52]. Poovazhahi & Thakur [53] reported that in active packaging, the balance between the concentration in the air space and the concentration on the food surface depends on the effect that determines the concentration of volatiles in the packaging system. The release of volatiles is directly proportional to the volatility of the antimicrobial agent. Food chemical composition also affects the absorption and their chemical reaction kinetics of the volatile compounds. Volatile antimicrobial agents are released from the carrier materials in gaseous form and occupy the entire free space within the packaging. This gas property facilitates the compound's distribution over the food, thus enhancing its antimicrobial activity [36].

### 3.2 Microbial modelling

**Fig. 3:**
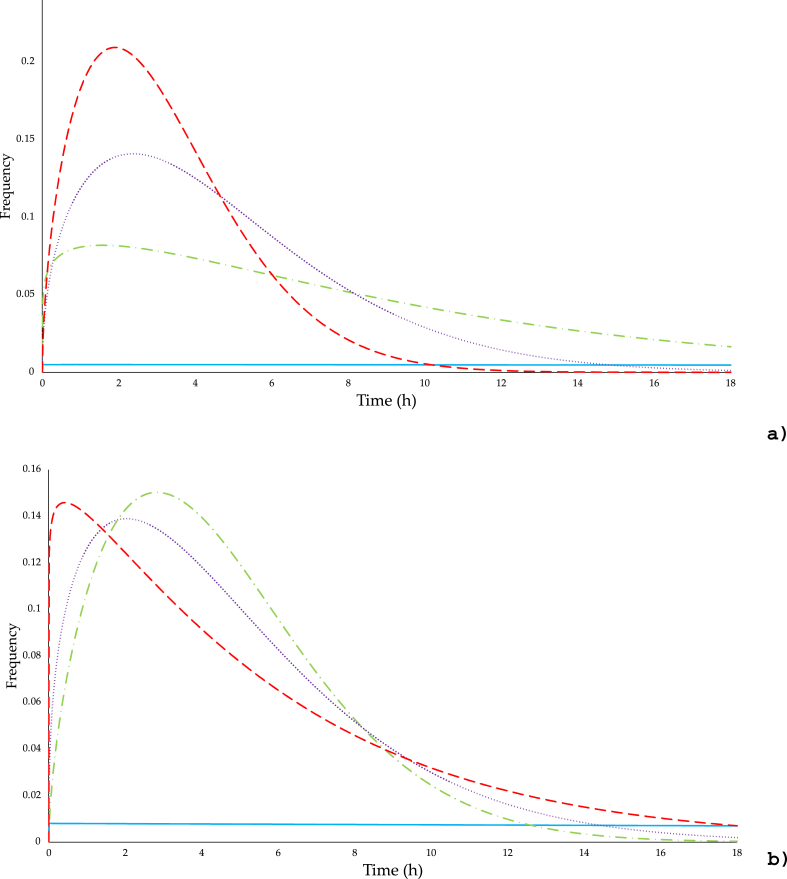
Frequency distribution of resistance of **a)***Escherichia coli* and **b)***Salmonella* serovar Typhimurium inoculated in a fresh salad during 48 h at 6 °C in different active packaging systems (Control **—,** 105 mg/L of air of carvacrol **-·-·-**,105 mg/L of air of thymol**···**, and a mixture of 52 mg/L of air of carvacrol and 52 mg/L of air of thymol **-** -).

The Weibull equation fitted all growth/inhibition curves with an average value of R2 = 0.973 ± 0.023. The parameters *b* and *n* for all experimental conditions are shown in Table 1. There are significant differences (p < 0.05) for both parameters in the antimicrobial mixture, demonstrating that it has the highest inhibitory effect on both inoculated microorganisms. Finally, the frequency analysis (Fig. 3) reveals that the mixture acts faster, but the effect differs. In the case of *E. coli* (Fig. 3A), the mixture reaches its maximum antimicrobial potential after 2 h and is much stronger than thymol and carvacrol. In the case of *Salmonella* (Fig. 3B), it is observed that the mixture reaches its maximum inhibitory power in less than 1 h, but with almost the same frequency values as the antimicrobials alone. Rojas et al. [35] mentioned that thymol and carvacrol are highly volatile components, and their loss during cast film solvent evaporation is inevitable. The formation of hydrophobic droplets further enhances their evaporation and yields the formation of voids in the polymer. Comparing both microorganisms, it can be said that *E. coli* is more resistant to the action of these components. Singh [26] noted that the antibacterial activity of the vapors depends on the type of compound and the duration of exposure. Moreover, disparities in antimicrobial activity may exist between compounds in the liquid state and those in the vapor phase. Thymol and carvacrol are oxygenated monoterpenes with stronger antimicrobial activities than oils, which are relatively rich in monoterpene hydrocarbons or sesquiterpenes [54]. These antimicrobial agents have a greater affinity for hydrophobic microbial cell membranes in the vapor phase due to their greater hydrophobicity. This increased cell membrane fluidity and permeability, disruption of membrane-embedded proteins, leakage of cytoplasm, inhibition of respiration, alteration of ion transport processes, permeability of bacterial cells due to their impregnation in the hydrophobic cell membrane, and a greater effect on Gram-positive bacteria [17,29]. In gram-negative bacteria, it also affects droplet formation, coagulation of cytoplasmic components, breakdown of cell structure, and absence of cytoplasmic material [14,19]. Firmanda et al. [19] also reported that the release rate of carvacrol increased with increasing temperature but was not affected by the humidity in package's headspace. Fancello et al. [55] indicated that antimicrobial activity may depend on the volatility of the active ingredients, which may increase the vapor capacity of the compounds. To enhance the release rate of active agents from polymer structures in food packaging, novel strategies like cocrystallization a multicomponent crystalline material aggregation can be employed. Rojas et al. [56] modified the physicochemical structures of eugenol in polylactic acid films, significantly altering the release profile of volatile compounds and enhancing its antimicrobial effect.

**Table 1:** Weibul model parameters (mean ± standard deviation) for *E. coli* and *Salmonella* serovar Typhimurium growth/inhibition curves inoculated in a fresh salad during 48 h at 6 °C in different active packaging systems (Control, 105 mg/L of air of carvacrol, 105 mg/L of air of thymol, and a mixture of 52 mg/L of air of carvacrol and 52 mg/L of air of thymol).

	*E. coli*			*Salmonella* serovar Typhimurium		
	*b*	*n*	R^2^	*b*	*n*	R^2^
Control	0.005 + 0.0003^a^	1.006 + 0.137^a^	0.9874	0.008 + 0.0027^a^	1.001 + 0.063^a^	0.9784
Carvacrol (105 mg/L of air)	0.076 + 0.0451^b^	−1.146 + 0.145^b^	0.9526	0.071 + 0.0091^b^	−1.624 + 0.087^b^	0.9434
Thymol (105 mg/L of air)	0.089 + 0.0098^c^	−1.458 + 0.319^b^	0.9692	0.102 + 0.0544^b^	−1.371 + 0.039^c^	0.9885
Carvacrol/Thymol (52/52 mg/L of air)	0.133 + 0.0027^d^	−1.571 + 0.082^c^	0.9815	0.154 + 0.0402^c^	−1.065 + 0.027^d^	0.9654

+ *b*: the rate of bacterial inactivation, and *n*, indicating how the microorganisms survive or die.

−Means followed by a different superscript letter within a column for each are significantly different (p < 0.05).

### 3.3 Consumer evaluation

**Fig. 4:**
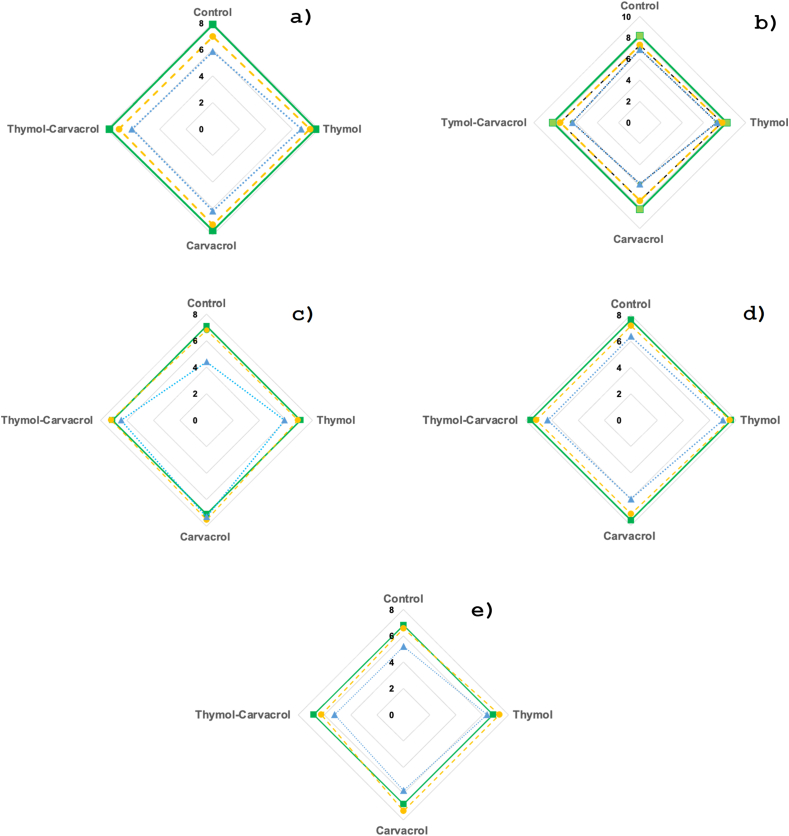
Consumer evaluation of a fresh salad: **a)** overall acceptability; **b)** color; **c)** odor; **d)** texture; and **e)** taste on a hedonic scale ranging from 1 to 9 at 12 days of storage (**-** day 0, **- -** day 6, **…**day 12) at 6 °C in different active packaging systems (Control; carvacrol 105 mg/L of air of carvacrol; 105 mg/L of air of thymol; and a mixture of 52 mg/L of air of carvacrol and 52 mg/L of air of thymol). (For interpretation of the references to color in this figure legend, the reader is referred to the Web version of this article.)

The results of the sensory analysis can be seen in Fig. 4. For all parameters evaluated, the effect of storage can be seen across all experiments; the ratings decreased from 8 to 6 on average. This could be because the salad components (lettuce and spinach) when cut into strips for presentation (Fig. 4D), exhibited changes in all sensory parameters studied (Fig. 4A). Regarding color (Fig. 4B), there is a natural degradation of chlorophyll, so the values are lowest on day 12. When evaluating the effect of antimicrobials on odor (Fig. 4C) and taste (Fig. 4E), no significant differences (p > 0.05) were observed between packages at the beginning of storage. However, at the end of miso storage, the control had the lowest scores (4.4), with significant differences (p < 0.05) compared to the other packages. Carvacrol and the thymol-carvacrol mixture scored the highest (7.3 and 6.4, respectively). As for taste at the end of storage, no significant differences were observed between them, although the average score was 5.4. The evaluators' comments did not reflect any dislike or finding of a strong taste characteristic of thymol and carvacrol. Similar results were reported by Delcarlo et al. [57] in taste and odor when adding oregano EO to fish fillets. All systems showed results surpassing the level of acceptability, and the mean scores of the systems containing the mixture of EOs were slightly higher than those of the control samples. Moreover, Lorenzo-Leal et al. [58] found that the application of EOs of thyme and rosemary in the vapor phase was more acceptable to consumers than the direct application of EOs. Terpenes like carvacrol and thymol have low levels of associated risks, making them as relatively safe compounds and earning them approval from regulatory authorities for use in food. Finally, Rathod et al. [27] reported that thymol exhibited slight oral toxicity, which increased with concentration (1000 mg/kg body weight), while carvacrol is non-toxic at lower levels. However, mild toxicity was reported at a higher concentration of 2480 mg/kg of body weight. Our concentrations used in these experiments were far from toxic levels.

## 4 Conclusions

In conclusion, our ﬁndings suggest that the combination of thymol and carvacrol has a synergistic effect on *E. coli* and *Salmonella* serovar Typhimurium when they are inoculated into a fresh green salad via the vapor phase. This combination has proven to be more efficient than the individual effect of these natural antimicrobials, thereby strengthening their antibacterial effects. Although the concentrations used in this paper did not affect sensory properties and are compatible with RTE vegetable salads, it is important to mention that this is an initial approach. Further information is necessary to enable its application as active packaging. Some challenges include the study of thymol and carvacrol on film structure and their controlled release, their molecule transfer from the active package through the food during storage time, the determination of shelf life, and toxicity, and the application of other techniques such as nanocompounds, cocrystallization, and nanoencapsulation, among others.

## Ethics statement

The sensory evaluation of the fresh salad samples were carried out in accordance with established ethical guidelines and informed consent was obtained from the participants. Participants were informed in advance of the purpose and the procedures of the study. Participants were assured of the confidentiality of their data. These experiments were carried out under established protocols and responsibility and commitment to the quality of the results obtained in this study is declared, ensuring that the procedures and methodologies used are in accordance with the relevant standards and regulations.

## Data availability statement

Data will be made available on request.

## CRediT authorship contribution statement

**Teresa Soledad Cid-Pérez:** Writing – original draft, Investigation, Data curation. **Ricardo Munguía-Pérez:** Writing – review & editing, Methodology, Investigation. **Guadalupe Virginia Nevárez-Moorillón:** Writing – review & editing, Validation, Methodology, Formal analysis. **Carlos Enrique Ochoa-Velasco:** Validation, Methodology, Conceptualization. **Addí Rhode Navarro-Cruz:** Writing – original draft, Validation, Methodology. **Raúl Avila-Sosa:** Writing – review & editing, Writing – original draft, Validation, Resources, Methodology, Investigation, Conceptualization.

## Declaration of competing interest

The authors declare the following financial interests/personal relationships which may be considered as potential competing interestsRaul Avila-Sosa reports administrative support was provided by Benemérita Universidad Autónoma de Puebla. Raul Avila-Sosa reports a relationship with Benemérita Universidad Autónoma de Puebla that includes: employment and non-financial support.
